# Genic distribution modelling predicts adaptation of the bank vole to climate change

**DOI:** 10.1038/s42003-022-03935-3

**Published:** 2022-09-16

**Authors:** Marco A. Escalante, Silvia Marková, Jeremy B. Searle, Petr Kotlík

**Affiliations:** 1 Laboratory of Molecular Ecology, Institute of Animal Physiology and Genetics, Czech Academy of Sciences, Liběchov, Czech Republic; 2 Department of Ecology and Evolutionary Biology, Cornell University, Ithaca, NY USA

**Keywords:** Ecological modelling, Population genetics

## Abstract

The most likely pathway for many species to survive future climate change is by pre-existing trait variation providing a fitness advantage under the new climate. Here we evaluate the potential role of haemoglobin (Hb) variation in bank voles under future climate change. We model gene-climate relationships for two functionally distinct Hb types, HbS and HbF, which have a north-south distribution in Britain presenting an unusually tractable system linking genetic variation in physiology to geographical and temporal variation in climate. Projections to future climatic conditions suggest a change in relative climatic suitability that would result in HbS being displaced by HbF in northern Britain. This would facilitate local adaptation to future climate—without Hb displacement, populations in northern Britain would likely be suboptimally adapted because their Hb would not match local climatic conditions. Our study shows how pre-existing physiological differences can influence the adaptive capacity of species to climate change.

## Abstract (web summary)

Haemoglobin variation in British bank voles combined with climate models predict future regional allelic replacement reflecting capacity for adaptation to climate change.

## Introduction

Rapid climate change predicted for the near future is likely to expose many species to conditions that exceed their climatic tolerance limits^1^. It is only species that respond by spatially tracking their climatic niches (range shifts) or by adapting to the new conditions within their current range that will avoid extinction^2^. Range shifts were a common response to past climate changes during the glacial-interglacial cycles of the Pleistocene, especially in the Northern Hemisphere^1,3,4^, and they are already increasingly observed in response to current climate change^5,6^. Range shifts are therefore likely to be an important response to climate change in the future^7^.

However, there are some situations where range shifts are not possible. This is most obviously the case for terrestrial species on oceanic islands^8^, but the same may apply to offshore islands^9^ or even the mainland for species with limited vagility^10^ or in fragmented habitats^11^, including species in mountains with limited opportunities for upslope migration^12^. Thus, it is only if there is in-situ adaptation that a species will continue to survive. The probability of emergence of new adaptive alleles is generally low, especially at loci with large effects that may be needed for adaptation to rapid climate change^13,14^. Therefore, pre-existing adaptive variation that happens to be able to provide a fitness advantage for new climatic conditions appears to be the most likely way that a species will persist during future climate change^1,15,16^. However, despite a wealth of evidence for the adaptation of populations of various species to local climatic conditions^17^, surprisingly little is known about the extent to which local adaptation patterns will be disrupted by future climate change, and how this may affect risk of extinction^11,18,19^.

Ecological niche modelling (ENM) has become a powerful tool for quantifying species-environment relationships and projecting the potential effects of climate change on species distribution and persistence^20,21^. These models generally assume that species are uniform with respect to climate-related traits, effectively ignoring local adaptation and the potential for species to adapt to climate change^22,23^. However, there have been some efforts to consider local adaptation in ENM by dividing species into genetic clusters associated with climatic conditions^24–26^. Such studies suggest that climate change is likely to disrupt patterns of gene-climate associations, with the severity of effects varying considerably among populations within the same species^25,26^. Insights beyond this into the mechanistic basis and limits of adaptation to climate change requires information about the genes underlying specific phenotypic traits relevant to climate adaptation^27^.

**Fig. 1:**
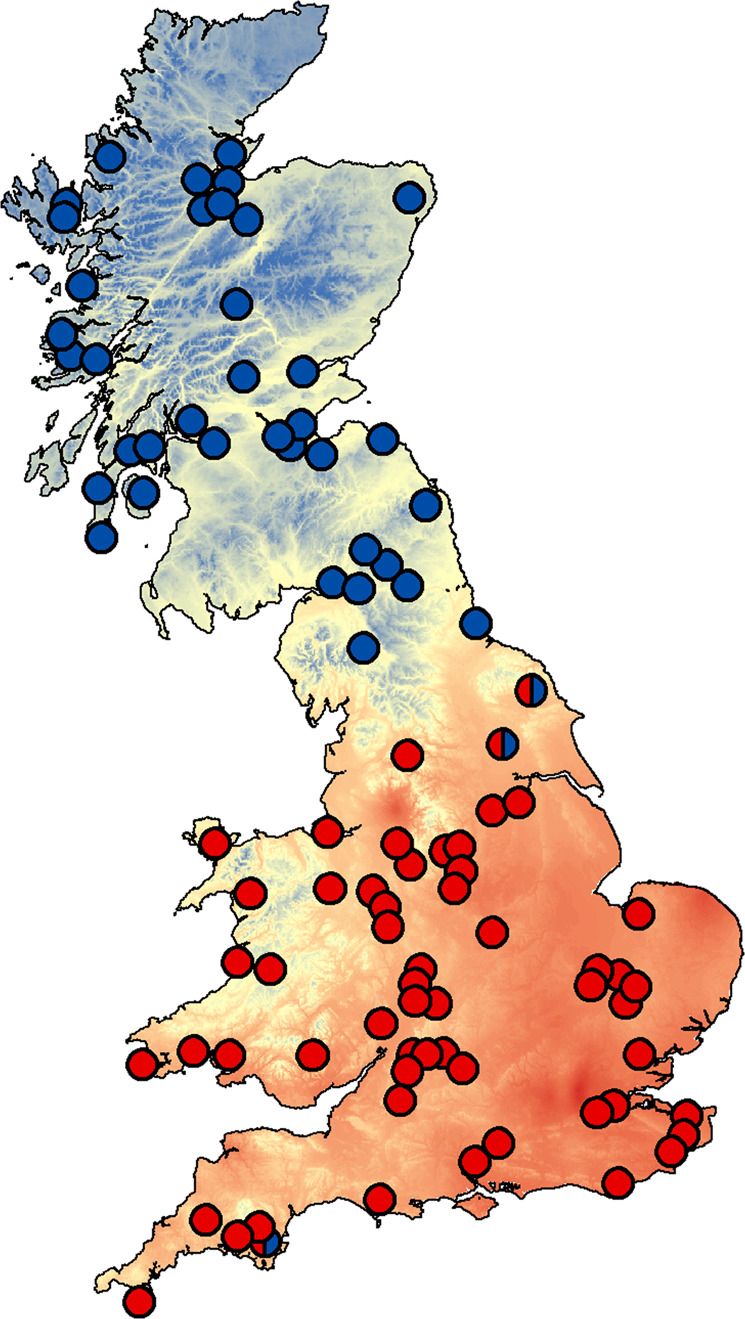
Geographic pattern of bank vole haemoglobin in Britain. Records of HbS are indicated by blue filling of the circles, those of HbF by red. The three sites where both HbS and HbF were detected are filled in half blue and half red. The background heat map shows the variation in mean summer temperature represented by the WorldClim variable BIO10^52^ (see text for details). The figure was created using ArcMap (v.10.8) and the Esri world countries dataset (www.esri.com).

Here, we evaluate the role of intraspecific physiological variation of haemoglobin (Hb) in the adaptation and survival of bank voles under future climate change. Bank voles (*Clethrionomys glareolus*) in Britain carry two functionally distinct Hb variants, HbS and HbF, which show a clear north-south pattern, with a boundary running through northern England^28,29^ (Fig. 1). This pattern can be explained by the arrival of firstly HbS, and secondly HbF, across the land bridge that connected England to mainland Europe until about 8500 years ago, with partial displacement of HbS by HbF^29,30^. The origin of HbS and HbF likely related to climatic conditions in glacial refugia, where bank voles survived the Last Glacial Maximum^29,31^. Haemoglobin is a well-studied, multifunctional molecule that has a major impact on adaptation to local climate in a wide variety of organisms^32,33^. Several lines of evidence suggest that Hb polymorphism of the bank vole represents local adaptation, as first proposed by Hall^28^. Bank vole HbS and HbF differ by a single amino acid substitution in the β-subunit, in which the serine β52 is replaced by a redox-active cysteine^29^. Vertebrate Hbs with redox-active cysteines play an important role in the antioxidant defence of erythrocytes^32,34–36^, and we have experimentally shown that HbF has a major effect in the bank vole by increasing the resistance of erythrocytes to oxidative stress^29^. Oxidative stress, triggered by a rise in environmental temperature, is increasingly recognized as a major source of selection pressure affecting the survivability and longevity of organisms during climate change^37–39^. On the other hand, the metabolic cost of HbF synthesis (due to limited Cys supply) is likely to result in lower fitness when it does not provide a major advantage, such as in more stable and colder environments^40^. Consistent with this, we have previously shown that the geographic distribution of HbF is related to climate variables representing annual and seasonal extremes, and that this relationship persists when adjusted for population structure^40^. Thus, the end-glacial replacement of HbS by HbF in southern Britain may have been a response to changing selection pressures associated with the climate warming at the time^29,30^, in which HbF allowed greater tolerance to environmental oxidative stress^29,39^. Therefore, Hb in bank voles in Britain represents an unusually tractable system linking genetic variation, which strongly affects climate-relevant physiological variation, to geographic and temporal climate variation.

To assess whether the presence of two Hb types, presumably adapted to different climatic conditions, facilitates the adaptability and survivability of bank vole populations in Britain under future climate change, we integrated Hb polymorphism into ENM. Specifically, we created climatic niche models for both HbS and HbF and defined the geographic regions that currently fall within the climatic range of each Hb type and those that will exhibit the same range in two future climate scenarios for the year 2070, one optimistic and one pessimistic^41^. The predicted shifts in climatic conditions indicate a change in relative climatic suitability that would result in HbS being displaced by HbF in northern Britain. This would facilitate local adaptation to future climate—without Hb displacement, populations in northern Britain would likely be suboptimally adapted because their Hb would not match local climatic conditions. These results demonstrate how pre-existing variation in physiological tolerance can influence the ability of populations and species to survive by adapting to climate change.

## Results and discussion

### Model performance and comparison

**Fig. 2:**
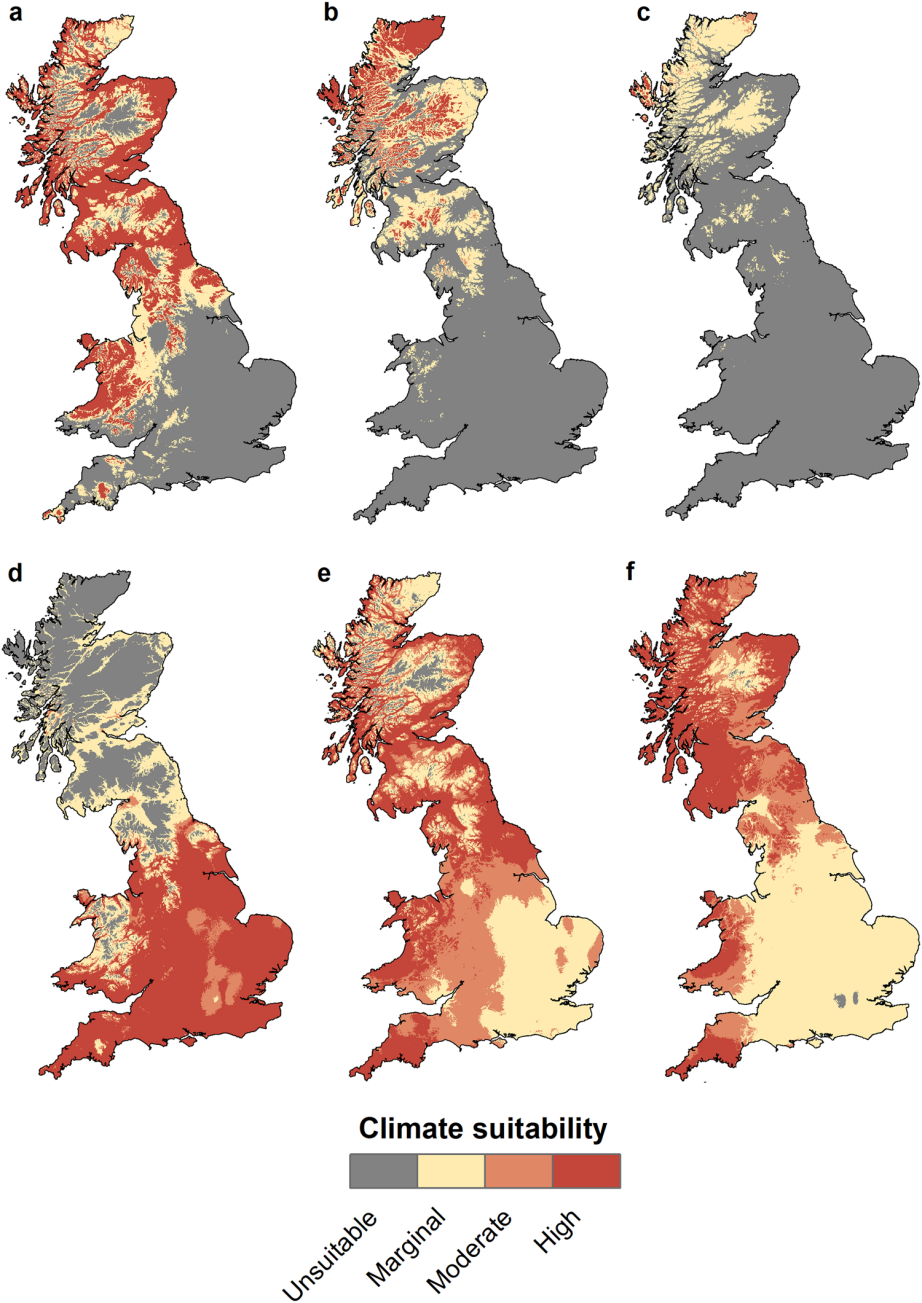
Predicted climate suitability for HbS and HbF. Panels **a**-**c** represent HbS, panels **d**-**f** represent HbF. **a**, **d** Present climate. **b**, **c**, **e**, **f** Optimistic (**b**, **e**) and pessimistic (**c**, **f**) future climate warming scenarios for 2070 (see text for details). The figure was created using ArcMap (v.10.8) and the Esri world countries dataset (www.esri.com).

All models built to define the climatic niches of HbS and HbF under current and future climate scenarios (Fig. 2) performed substantially better than expected by chance, both by area under the curve (AUC = 0.79–0.88)^42^ and by partial AUC (pAUC > 1)^43^, with AUC values indicating good to excellent performance of the models^42^ (Supplementary Table 1).

The overlap between the climatic niches occupied by HbS and HbF is significantly smaller than expected by chance (randomization tests, *n* = 100, *p* < 0.001), both in the geographic space defined by the climatic conditions currently prevailing in Britain (Schoener’s *D* = 0.28) and in a continuous multidimensional space of climatic variables (*D* = 0.34 and *D* = 0.37 for two different sets of climatic variables, Set 1 and Set 2, respectively) (Supplementary Table 2 and Supplementary Fig. 1). This suggests that the climatic differences between the distribution areas of HbS and HbF are due to different climatic tolerances between the two Hb types, rather than equal tolerance of both Hb types and whatever climatic differences between the areas where each Hb type happens to occur^44,45^.

The climatic variables with the highest contributions to the models were related to both temperature and precipitation (Supplementary Fig. 2). The most important variable for all models was the mean temperature of the warmest quarter (BIO10, hereafter called mean summer temperature; Fig. 1). For HbS, there was an abrupt decrease in climate suitability at sites with a mean temperature of the warmest quarter above 14 °C, while for HbF the optimum was above 15 °C. Compared to HbF, suitability for HbS was higher at sites with relatively lower minimum temperature of the coldest month (BIO6) and with lower mean temperature of the coldest quarter (BIO11). HbS was also positively affected by annual precipitation (BIO12) and precipitation of the wettest month (BIO13), whereas an opposite relationship was observed for HbF. Finally, HbF was positively influenced by the seasonality of temperature (BIO4) and negatively influenced by the seasonality of precipitation (BIO15), while HbS was negatively influenced by the former (BIO4) and positively influenced by the latter (BIO15).

### Present and future climate suitability

**Fig. 3:**
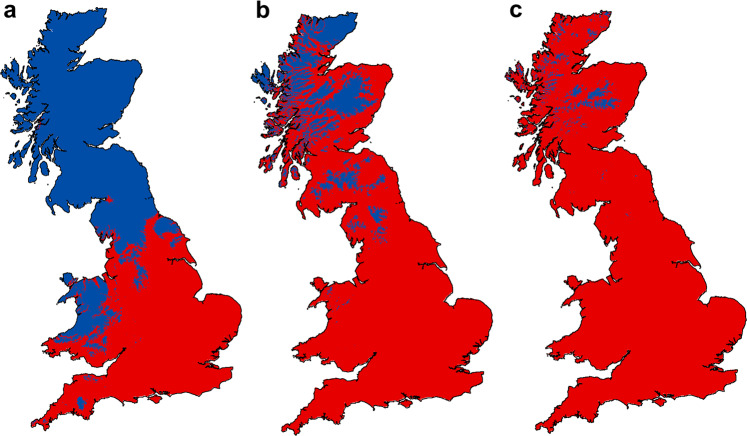
Relative climate suitability for HbS and HbF. Blue colour indicates areas where climate is predicted to favour HbS over HbF, red colour indicates areas where climate is predicted to favour HbF over HbS. **a** Present climate. **b**, **c** Optimistic (**b**) and pessimistic (**c**) future climate scenarios. The figure was created using ArcMap (v.10.8) and the Esri world countries dataset (www.esri.com).

The spatial projections of the models to the present conditions (Fig. 2a, d) showed relative climatic suitability for HbS and HbF (Fig. 3a) consistent with the distribution of occurrence points for each Hb type (Fig. 1). The results obtained with two different sets of climate variables (Set 1 and Set 2) for comparison are very similar (Figs. 2 and 3 and Supplementary Figs. 3, 4), and we present only the results based on Set 1 in the text and Figs. 2 and 3. According to these results, HbS is favoured over HbF in about 50% of Britain. Consistent with its current distribution, HbS is favoured in most parts of Scotland and northern England (Fig. 3a), where there are generally cold winters, cool summers, and high annual rainfall^46^. In addition, the northern part of Wales, where HbS has not been recorded (Fig. 1), is also predicted to have a favourable climate for HbS. In contrast, HbF is favoured in most parts of England (Fig. 3a), which are generally characterised by warm summers, mild to cold winters, and relatively low annual rainfall^46^.

While the model projections generally agree well with the Hb distribution pattern over Britain, there is a discrepancy in Wales. We cannot rule out the possibility that HbS escaped sampling in Wales, as there are local climates in Wales that favour both HbS and HbF (Fig. 3a). However, another possibility for why HbS does not occur in a climatically suitable area surrounded by HbF is that it was displaced when conditions favoured HbF and there was no opportunity for it to re-enter Wales thereafter.

A notable difference in climate suitability between HbS and HbF is observed when the models are projected to future climate (Fig. 2b, c, e, f). A climate that is highly suitable for putatively cold-adapted HbS is confined to the Highlands and the northern fringes of Scotland by 2070 (Fig. 2b, c), especially in the pessimistic scenario (Fig. 2c). Instead, much of Scotland is predicted to have a future climate that is highly suitable for putatively warm-adapted HbF (Fig. 2e, f). Consequently, HbF is predicted to be the favoured Hb type for more than 80% of Britain in the optimistic (RCP 2.6) climate warming scenario and for more than 97% of the country in the pessimistic (RCP 8.5) scenario (Fig. 3b, c).

### Adaptation to future climate change

The changes in relative climatic suitability suggest that there will be positive selection pressure for allelic turnover in populations of bank vole in northern Britain. Assuming gene flow rates are high enough^25^, in such a scenario the poorly adapted HbS would be displaced by the warm-adapted HbF in much of Scotland and northern England and would survive only in limited areas, especially under the pessimistic climate change scenario (Fig. 3). This turnover between HbS and HbF would facilitate adaptation of northern bank vole populations to new climatic conditions. We cannot rule out the possibility that some populations will go extinct, but it seems more likely that migration of HbF from south to north will allow northern populations to adapt. The bank vole is widespread in bushy edge habitats in Britain, and its population structure is not particularly unusual for a small mammal^47^, suggesting that there are no strong barriers to north-south dispersal of HbF. Such a future scenario would be consistent with the displacement of HbS by HbF in southern Britain during end-glacial climate warming^29,30^.

Our models also predict that climatic suitability for HbF in much of England will change from highly suitable to only marginally suitable under both future scenarios (Fig. 2d–f), although it will remain the favoured Hb type in the comparison of HbS and HbF (Fig. 3). However, caution must be exercised in interpretation here, as predicting adaptation to a climate beyond the range of temperatures to which voles are currently exposed could lead to poor predictive accuracy^48^. For example, mean summer temperatures (BIO10) in Britain are currently between 8–18 °C and, according to the pessimistic scenario, will rise to 10–22 °C by 2070^41^. Therefore, it is not possible to say with certainty how well voles with HbF will be adapted to warmer conditions than those currently experienced in Britain. The low suitability of HbF in southern Britain under future climate scenarios may therefore be in part an artefact of the way the models were constructed.

Whatever the situation in southern Britain, our results are clear in suggesting that existing Hb variation in Britain will play an important role in bank vole adaptation to future climate change in the north of the landmass. Predictions from our models suggest that displacement of HbS by HbF is important for northern populations to adapt to future climate conditions. Without this displacement, the Hb of bank voles in much of Scotland would likely be poorly adapted because it would not match local climate conditions^48^. Our study thus highlights the key role of pre-existing genetic variation—the presence of HbS and HbF in Britain—in the evolutionary adaptation of the physiology of a widespread small mammal species to future climate change.

## Conclusions

Identifying climate-relevant functional variation remains a challenge, especially in non-traditional model species, so most ENM studies to date have relied on loci of unknown functional/physiological relevance^48^. In the present study, we have taken advantage of bank vole Hb, which is an unusually tractable system that links genetic variation in climate-relevant physiological tolerance to geographic and temporal climate variation^29,40^. We have shown that this Hb variation may provide bank vole populations with important adaptive opportunities to future climate change. The next step is to validate the relationship between Hb polymorphism and bank vole fitness under different environmental conditions using experimental approaches^31^. We have already shown that HbF has an important physiological effect in the bank vole by significantly increasing cellular antioxidant capacity^28^. However, it is important to keep in mind that the endogenous antioxidant system is complex and the cysteine-based Hb and glutathione system, although the most important, represents only one of several lines of defence against reactive oxygen species^35^, all of which may influence the response to selection. Thus, our approach necessarily represents a simplification of the complex evolutionary and ecological processes that influence population and species adaptation. Despite this limitation, however, our study is an example of how pre-existing genetic differences in physiological tolerance can influence the adaptive capacity of populations to changing climatic conditions, and it underscores the need to consider such differences when predicting the effects of future climate change. This is the necessary step toward understanding the mechanistic basis and limits of local adaptation^27^, and ultimately toward more realistic models to explain and predict the survival potential of populations and species in a changing climate^23,49^.

## Methods

### Haemoglobin data

For the geographic distribution of HbS and HbF in Britain, we combined published data^28,29^ with new data for previously available bank vole samples^29,30^ in which we determined Hb type by genotyping the polymorphic codon 52 site that distinguishes HbS and HbF by Sanger sequencing of PCR amplicons of the HBB-T1 beta-globin gene at Macrogen Inc.^29,40^. We selected sampling points that were at least 10 km apart to reduce spatial autocorrelation in climate data^50^. This resulted in a total of 94 occurrence data points for ENM, 40 for HbS and 57 for HbF (three points were common to both types; Fig. 1; Supplementary Table 3).

### Model building, tuning and validation

We used MaxEnt v.3.4.1^51^ with present-day climate data and background sample points randomly selected within the area of Britain to build climatic niche models for HbS and HbF. We used two subsets of 19 bioclimatic variables available in the WorldClim v.1.4 dataset^52^ at 30 arc-second resolution. Each set was selected through a reiterative jackknife process^53^, but in the first case (Set 1) HbS occurrences were used as training data, while in the second case (Set 2) the models were trained with HbF dataset (see Supplementary Methods for details). After removing highly correlated variables (*r* > 0.8), Set 1 included temperature seasonality (BIO4), mean temperature of the warmest quarter (BIO10), precipitation of the wettest month (BIO13), and precipitation seasonality (BIO15), while the variables in Set 2 were minimum temperature of the coldest month (BIO6), mean temperature of the warmest quarter (BIO10), mean temperature of the coldest quarter (BIO11), and annual precipitation (BIO12).

After refining the MaxEnt parameters using the AICc approach^54^ implemented in ENMTools v.1.4^55^, two separate models were built for each set of variables (Set 1 and Set 2): one for HbS and one for HbF. A total of 50 replicates of each model were generated by the subsampling method in MaxEnt, in which test data points are randomly selected for cross-validation of the model^51^. Subsequently, estimates were based on the mean of the replicates (see Supplementary Methods for details).

### Model projection and comparison

To predict future shifts in climate suitability for HbS and HbF, the models were projected to a range of climate scenarios for the year 2070 (average for 2061-2080) driven by four general circulation models (GCMs) for future climate: CCSM4^56^, IPSL-CM5A-LR^57^, MIROC-ESM^58^, and MPI-ESM^59^, with each model considering two representative concentration pathways (RCPs) corresponding to different predicted levels of greenhouse gas emissions, representing an optimistic (RCP 2.6) and a pessimistic (RCP 8.5) scenario in terms of temperature increase^41^. RCP 2.6 represents an increase in global temperature between 0.3 and 1.7 °C by 2100, while RCP 8.5 indicates an increase in global temperature between 2.6 and 4.8 °C^41^.

To quantify the overlap of climate niche for HbS and HbF, we used Schoener’s *D* metric^44,60^, ranging from 0 (no overlap) to 1 (complete overlap), and tested the significance of the overlap to evaluate the hypothesis of niche identity between HbS and HbF by randomizing the occurrence points 100 times between the two Hb types with ENMTools R package v.1.0^44,45^. Niche overlap was calculated based on predicted suitability in the geographic space of climatic conditions prevailing in Britain^44^ and in continuous multidimensional climatic space^22,45^. We also performed a test for ‘background similarity’ to assess whether the relationship of HbS and HbF with different climates could be explained solely by climatic differences between the regions of Britain occupied by each type^44,45^. The null distribution of overlap values for this test was generated by drawing a set of random background points 100 times for each Hb type (see Supplementary Methods for details).

To summarize the results across the four GCMs, the future projections were averaged independently for each combination of a set of variables (either Set 1 or Set 2) and climate warming scenario (either RCP 2.6 or RCP 8.5). To define the geographic regions that currently fall within the climatic range of HbS and HbF, and those that will have the same range in the future, we created climate suitability maps based on three different thresholds: the minimum training presence (marginal suitability), the fifth percentile training presence (moderate suitability), and the 10th percentile training presence (high suitability). To illustrate the change in relative climatic suitability of HbS and HbF under projected climate change, we created maps showing which Hb type is favoured at any location under current and future climate. All the maps were created using ArcMap (v.10.8) and the Esri world countries dataset (www.esri.com).

### Statistics and reproducibility

To ensure reproducibility of the results, 50 replicates of each model were built with MaxEnt v.3.4.1. Randomization tests of niche identity (one-sided) and background similarity (two-sided) were performed with 100 replicates in ENMTools R package v.1.0.

### Reporting summary

Further information on research design is available in the Nature Research Reporting Summary linked to this article.

## Supplementary information

Supplementary Information

Reporting Summary

## Data Availability

The haemoglobin genotype occurrence data generated and analysed in this study are included in the supplementary information file. WorldClim climate data are publicly available at www.worldclim.org. Any additional information can be obtained directly from the authors on reasonable request.

## Code availability

No previously unreported custom computer code was used to produce the results in this study.
