# Supplementary material for: Genic distribution modelling predicts adaptation of the bank vole to climate change
Source: Commun Biol. 2022 Sep 16;5:981. doi: 10.1038/s42003-022-03935-3 (PMC9481625; doi:10.1038/s42003-022-03935-3)
Supplement: Supplementary file 1 — Supplementary Information [file 42003_2022_3935_MOESM1_ESM.pdf]

Supplementary Information

Genic distribution modelling predicts adaptation of the bank vole to climate change

Marco A. Escalante, Silvia Marková, Jeremy B. Searle & Petr Kotlík

This PDF file includes:
Supplementary Methods
Supplementary References
Supplementary Tables 1–3
Supplementary Figures 1–4

Supplementary Methods

Selection of bioclimatic variables

Two different sets of predictor variables were used for comparison. Both sets were selected based on a reiterative jackknife process of model construction and stepwise removal of the least contributing variables[1]. BIO8, BIO9, BIO18, and BIO19 were removed *a priori* from the full set of 19 variables due to known spatial artefacts[2]. To create the first set of predictors (Set 1), the HbS occurrences were used as the training dataset. The contribution of each variable to the model was then determined using a jackknife approach in MaxEnt, and the variable contributing least to the model was removed from the training dataset at each iteration. A new model was then created using the remaining variables and the process was repeated until a set of variables with the highest contribution to the model was found. The final set of variables was then created by removing one variable from each pair of correlated variables in a correlation matrix of climate layers calculated in ArcMap (cut-off of $r < 0.8$; ref. [3]). The variables included in Set 1 were temperature seasonality (BIO4), mean temperature of the warmest quarter (BIO10), precipitation of the wettest month (BIO13), and precipitation seasonality (BIO15). The second set of variables (Set 2) was created using the same procedure, but using the HbF occurrences as the training dataset. The variables included in Set 2 were minimum temperature of the coldest month (BIO6), mean temperature of the warmest quarter (BIO10), mean temperature of the coldest quarter (BIO11), and annual precipitation (BIO12).

MaxEnt tuning and model validation

To fine-tune the MaxEnt parameters, we considered seven combinations of feature types (i.e., Linear, Hinge, Linear + Quadratic, Linear + Quadratic + Hinge, Linear + Quadratic + Product, Linear + Quadratic + Hinge + Product, and Linear + Quadratic + Hinge + Product + Threshold) to calculate the probability distribution[4] and evaluated six regularization multipliers (1, 2, 5, 10, 15, 20) based on previously described criteria[5–7]. A total of 42 models with different combinations of features and regularization parameters were independently tested for HbS occurrences with Set 1 and for HbF occurrences with Set 2. In both cases, the best parameter combination was selected using the AICc approach[5] implemented in ENMTools 1.4[8]: Feature Type = Hinge and Regularization Multiplier = 2.

After parameter tuning, two separate models were created with each set of variables (Set 1 and Set 2), one for HbS and one for HbF. A total of 50 replicates of each model were generated by the subsampling method in MaxEnt, which randomly selects 25% of the occurrence points reserved as test data[9]. To minimize the potential impact of inadequate ecological background representation, 1,000,000 background points were used[10]. Model performance was evaluated using the average area under the receiver operating characteristic curve (AUC) for the test data as a standard measure of predictive ability[11]. The partial AUC ratio (pAUC; ref. [12]) was also calculated using the Niche Tool Box[13].

Tests of niche similarity

Niche comparisons were performed using the ENMTools R package[14] independently for Set 1 and Set 2. Niche overlap was quantified using Schoener's *D*[15,16] on a scale from 0 (no overlap) to 1 (identical niche models). Niche overlap was calculated based on predicted suitability in geographic space (*G*-space) determined by combinations of climatic variable values available across Britain[15], and for a random sample from the continuous multidimensional space of climatic variables (*E*-space) whose axes are bounded by the minimum and maximum values of the respective climate grids[14].

Niche identity tests were applied to assess whether the niche models for the two Hb types could be considered equivalent[14,15]. To generate the expected null distributions, the occurrences of the two Hb types were pooled and their identities randomised to generate two new samples with the same number of observations as the empirical datasets. This was repeated 100 times for each pair of Hb types. The hypothesis that the niches are identical is rejected if the empirical *D* value is significantly lower (one-sided test) than the expected null distribution (at the 0.05 level).

In addition, the background similarity test was used to assess whether the niches for the two Hb types are more similar or different than would be expected by chance given the environmental differences between the regions where the Hb types occur[14,15,17]. This two-sided test compares the observed value of the niche overlap metric to a null distribution expected between a random sample of points from the range of the two Hb types. If the observed value of the niche overlap is significantly lower (indicating more different niches than expected by chance) or higher (indicating more similar niches than expected by chance) than expected from 100 pseudo-replicates, the null hypothesis that the difference between Hb types is no different than expected based on available climatic differences is rejected. To test whether the Hb types occupy niches that are more different than would be expected by chance based on the available climates, and to examine the effect of background choice, two different analyses were performed. First, the distribution of each Hb type was used as background (i.e. the northern part of Britain for HbS and the southern part of Britain for HbF), and second, the whole of Britain was used as background for both Hb types. Identity and background tests were performed in both *G*-space and *E*-space as defined above.

Supplementary References

1. Zeng, Y., Low, B. W. & Yeo, D. C. J. Novel methods to select environmental variables in MaxEnt: A case study using invasive crayfish. *Ecol. Modell.* **341**, 5–13 (2016).
2. Ashraf, U. *et al.* Ecological niche model comparison under different climate scenarios: a case study of *Olea* spp. in Asia. *Ecosphere* **8**, e01825 (2017).

3. Merow, C., Smith, M. J. & Silander, J. A. A practical guide to MaxEnt for modeling species' distributions: what it does, and why inputs and settings matter. *Ecography* **36**, 1058–1069 (2013).
4. Li, Y., Li, M., Li, C. & Liu, Z. Optimized maxent model predictions of climate change impacts on the suitable distribution of *Cunninghamia lanceolata* in China. *Forests* **11**, 302 (2020).
5. Warren, D. L. & Seifert, S. N. Ecological niche modeling in Maxent: The importance of model complexity and the performance of model selection criteria. *Ecol. Appl.* **21**, 335–342 (2011).
6. Shcheglovitova, M. & Anderson, R. P. Estimating optimal complexity for ecological niche models: A jackknife approach for species with small sample sizes. *Ecol. Modell.* **269**, 9–17 (2013).
7. Morales, N. S., Fernández, I. C. & Baca-González, V. MaxEnt's parameter configuration and small samples: are we paying attention to recommendations? A systematic review. *PeerJ* **5**, e3093 (2017).
8. Warren, D. L., Glor, R. E. & Turelli, M. ENMTools: A toolbox for comparative studies of environmental niche models. *Ecography* **33**, 607–611 (2010).
9. Phillips, S. J., Dudík, M. & Schapire, R. E. A maximum entropy approach to species distribution modeling. in *Twenty-first International Conference on Machine Learning - ICML '04* **9**, 83 (ACM Press, 2004).
10. Guevara, L., Gerstner, B. E., Kass, J. M. & Anderson, R. P. Toward ecologically realistic predictions of species distributions: A cross-time example from tropical montane cloud forests. *Glob. Chang. Biol.* **24**, 1511–1522 (2018).
11. Guisan, A. & Zimmermann, N. E. Predictive habitat distribution models in ecology. *Ecol. Modell.* **135**, 147–186 (2000).
12. Peterson, A. T., Papeş, M. & Soberón, J. Rethinking receiver operating characteristic analysis applications in ecological niche modeling. *Ecol. Modell.* **213**, 63–72 (2008).
13. Osorio-Olvera, L. *et al.* ntbox: An r package with graphical user interface for modelling and evaluating multidimensional ecological niches. *Methods Ecol. Evol.* **11**, 1199–1206 (2020).
14. Warren, D. L., Beaumont, L. J., Dinnage, R. & Baumgartner, J. B. New methods for measuring ENM breadth and overlap in environmental space. *Ecography* **42**, 444–446 (2019).
15. Warren, D. L., Glor, R. E. & Turelli, M. Environmental niche equivalency versus conservatism: Quantitative approaches to niche evolution. *Evolution* **62**, 2868–2883 (2008).
16. Schoener, T. W. The anolis lizards of Bimini: resource partitioning in a complex fauna. *Ecology* **49**, 704–726 (1968).
17. Warren, D. L. *et al.* ENMTools 1.0: an R package for comparative ecological biogeography. *Ecography* **44**, 504–511 (2021).
18. Hall, S. J. G. Haemoglobin polymorphism in the bank vole, *Clethrionomys glareolus*, in Britain. *J. Zool.* **187**, 153–160 (1979).
19. Kotlík, P. *et al.* Adaptive phylogeography: Functional divergence between haemoglobins derived from different glacial refugia in the bank vole. *Proc. R. Soc. B* **281**, 20140021 (2014).

Supplementary Table 1. Performance of ecological niche models build using two different sets of predictor (climatic) variables (Set 1 and Set 2), evaluated by the average test area under the curve (AUC), partial AUC ratio calculated at 0% omission rate (pAUC).

**Predictors**	**Model**	**Occurrences**	**Test AUC (SD)**	**pAUC (SD)**
Set 1	HbS	40	0.88 (0.02)	1.73 (0.1)
	HbF	57	0.86 (0.02)	1.72 (0.09)
Set 2	HbS	40	0.8 (0.03)	1.58 (0.1)
	HbF	57	0.79 (0.03)	1.55 (0.1)

Supplementary Table 2. Tests of niche identity between Hb types calculated based on niche models built with two different sets of predictor (climatic) variables (Set 1 and Set 2). Niche overlap, quantified by Schoener's *D*, is evaluated in geographical (*G*) as well as environmental (*E*) space. A significant identity test indicates that Hb types show measurable differences in niche occupancy.

			**Model A versus Model B**	
**Predictors**	**Model A**	**Model B**	***G*-space**	***E-space***
Set 1	HbS	HbF	0.28***	0.34***
Set 2	HbS	HbF	0.28***	0.37***

***Niches significantly different (one-sided randomization test, n = 100, P < 0.001)

Supplementary Table 3. Bank vole haemoglobin (Hb) type occurrences in Britain. Published data by Hall (1979)[18] and Kotlík et al. (2014)[19] were combined with new data obtained from previously available bank vole samples, collected largely between 1990 and 2011.

Locality	Latitude	Longitude	Hb	Source
Almondell	55.91	-3.46	HbS	ref. [18]
Portavadie	55.88	-5.31	HbS	ref. [18]
Chillingham	55.52	-1.90	HbS	ref. [18]
Castle Eden Dene	54.74	-1.32	HbS	ref. [18]
Morland	54.58	-2.62	HbS	ref. [18]
Wycoler	53.85	-2.11	HbF	ref. [18]
Wirral	53.33	-3.03	HbF	ref. [18]
Anglesey	53.25	-4.33	HbF	ref. [18]
Snowdonia	52.90	-3.92	HbF	ref. [18]
Aberystwyth	52.42	-4.07	HbF	ref. [18]
Wicken Fen	52.33	0.33	HbF	ref. [18]
Dale	51.70	-5.17	HbF	ref. [18]
Holne	50.52	-3.82	HbF	ref. [18]
Lizard	49.97	-5.20	HbF	ref. [18]
Maud	57.48	-2.08	HbS	ref. [19]
Raasay	57.44	-6.04	HbS	ref. [19]
Raasay	57.36	-6.07	HbS	ref. [19]
Mull	56.49	-5.99	HbS	ref. [19]
Roslin	55.85	-3.10	HbS	ref. [19]
Pickering	54.29	-0.68	HbS/HbF	ref. [19]
York	53.93	-1.01	HbS/HbF	ref. [19]
Doncaster	53.47	-1.13	HbF	ref. [19]
Temple End	52.13	0.41	HbF	ref. [19]
Cirencester	51.78	-1.69	HbF	ref. [19]
Cirencester	51.77	-2.05	HbF	ref. [19]
Cirencester	51.77	-1.89	HbF	ref. [19]
North Hill	50.57	-4.44	HbF	ref. [19]
Diptford	50.41	-3.75	HbS/HbF	ref. [19]
Invergordon	57.75	-4.15	HbS	new
Poolewe	57.73	-5.54	HbS	new
Strathpeffer	57.59	-4.53	HbS	new
Avoch	57.57	-4.17	HbS	new
Torbreck	57.44	-4.25	HbS	new
Kiltarlity	57.40	-4.46	HbS	new
Tomatin	57.34	-3.96	HbS	new
Bunacaimb	56.93	-5.86	HbS	new
Perthshire	56.81	-4.08	HbS	new
Tobermory	56.62	-6.10	HbS	new
Craignure	56.46	-5.70	HbS	new
Glencarse	56.38	-3.31	HbS	new
Glen Artney	56.35	-4.00	HbS	new
Loch Lomond	56.07	-4.60	HbS	new
Dalgety Bay	56.04	-3.34	HbS	new
Linlithgow	55.97	-3.60	HbS	new
Thorntonloch	55.95	-2.39	HbS	new
Bearsden	55.92	-4.34	HbS	new

Locality	Latitude	Longitude	Hb	Source
Cowal	55.90	-5.08	HbS	new
Tayinloan	55.63	-5.67	HbS	new
Brodick	55.60	-5.15	HbS	new
Southend	55.31	-5.64	HbS	new
Kielder Forest	55.23	-2.59	HbS	new
Kielder Forest	55.13	-2.34	HbS	new
Longtown	55.01	-2.97	HbS	new
Hexham	54.99	-2.11	HbS	new
Brampton	54.97	-2.67	HbS	new
Epworth	53.53	-0.82	HbF	new
Monk's Heath	53.25	-2.23	HbF	new
Derbyshire	53.20	-1.68	HbF	new
Longsdon	53.10	-2.07	HbF	new
Ambergate	53.06	-1.49	HbF	new
Street Dinas	52.94	-3.00	HbF	new
Ashbourne	52.94	-1.57	HbF	new
Longslow	52.92	-2.51	HbF	new
Weston Jones	52.81	-2.37	HbF	new
Grimston	52.76	0.56	HbF	new
Tong Forge	52.67	-2.33	HbF	new
Leicester area	52.63	-1.13	HbF	new
Aberystwyth	52.37	-3.69	HbF	new
Hopwood	52.37	-1.96	HbF	new
Cambridgeshire	52.36	0.08	HbF	new
Littleworth	52.27	-2.02	HbF	new
Cambridge	52.26	0.01	HbF	new
Gazeley	52.25	0.52	HbF	new
Sheffield	53.23	-1.51	HbF	new
Throckmorton	52.15	-2.02	HbF	new
Long Marston	52.13	-1.78	HbF	new
Ryton	51.99	-2.41	HbF	new
Llanddowror	51.79	-4.58	HbF	new
Essex	51.77	0.57	HbF	new
Gwent	51.76	-3.20	HbF	new
Pontyberem	51.76	-4.16	HbF	new
Pusey	51.67	-1.47	HbF	new
Ashley	51.65	-2.11	HbF	new
Corsham	51.44	-2.19	HbF	new
Kent	51.40	0.27	HbF	new
Bromley	51.35	0.08	HbF	new
Herne Common	51.33	1.11	HbF	new
Bradley Hill	51.19	1.08	HbF	new
Medstead	51.13	-1.06	HbF	new
Bonnington	51.07	0.94	HbF	new
Brambridge	51.00	-1.33	HbF	new
Pevensey	50.84	0.32	HbF	new
Dorset	50.72	-2.43	HbF	new
Bickleigh	50.45	-4.07	HbF	new

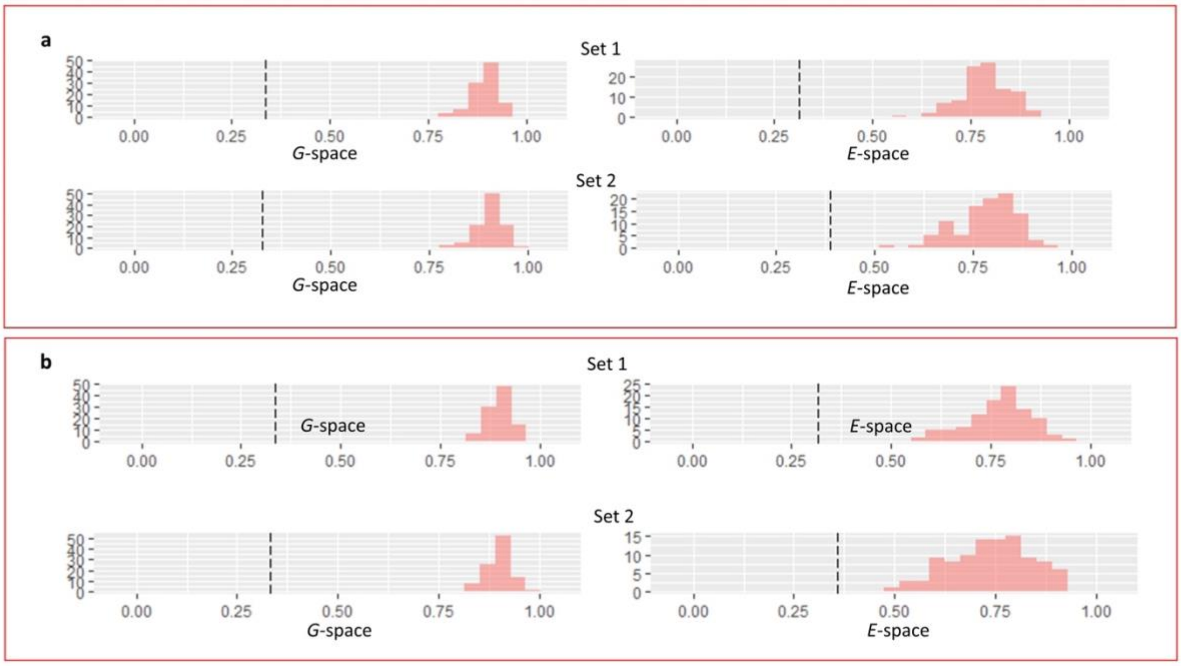

Supplementary Figure 1. Background similarity test comparing the observed similarity of the climatic niches for HbS and HbF (quantified by the *D* value; vertical dashed line) with the null distribution of similarities (pink bars) generated based on a random sample of points from the distribution range of each Hb type. The null hypothesis is rejected if the observed *D* value falls outside the 95% confidence limits of the null distribution (two-sided test). All the tests are significant (two-sided randomization test, n = 100, P < 0.001) suggesting that the climatic differences between HbS and HbF are due to a divergence in their climatic tolerance, rather than an equal tolerance, but differences in subsets of the favourable climates available to each[15,17]. The distribution of each Hb type (a) and all of Britain (b) were considered independently as background areas.

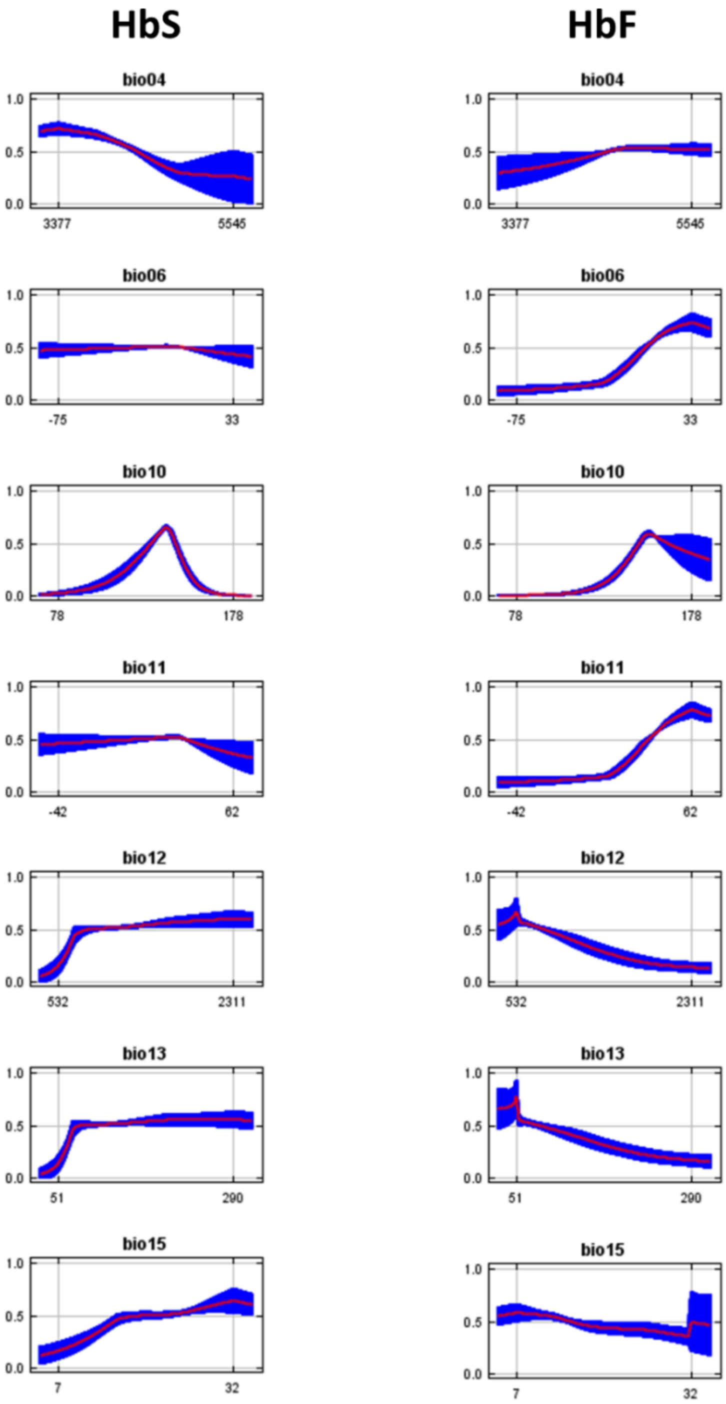

Supplementary Figure 2. Response curves illustrating the relationship between MaxEnt predicted climate suitability and the climatic variables included in Set 1 and Set 2: temperature seasonality (BIO4), minimum temperature of the coldest month (BIO6), mean temperature of the warmest quarter (BIO10), mean temperature of the coldest quarter (BIO11), annual precipitation (BIO12), precipitation of the wettest month (BIO13), and precipitation seasonality (BIO15). The x-axis represents the range of each variable in Britain, while the y-axis indicates the predicted climate suitability. The curves show the mean response of the 50 replicate Maxent runs (red) and the mean +/- one standard deviation (blue).

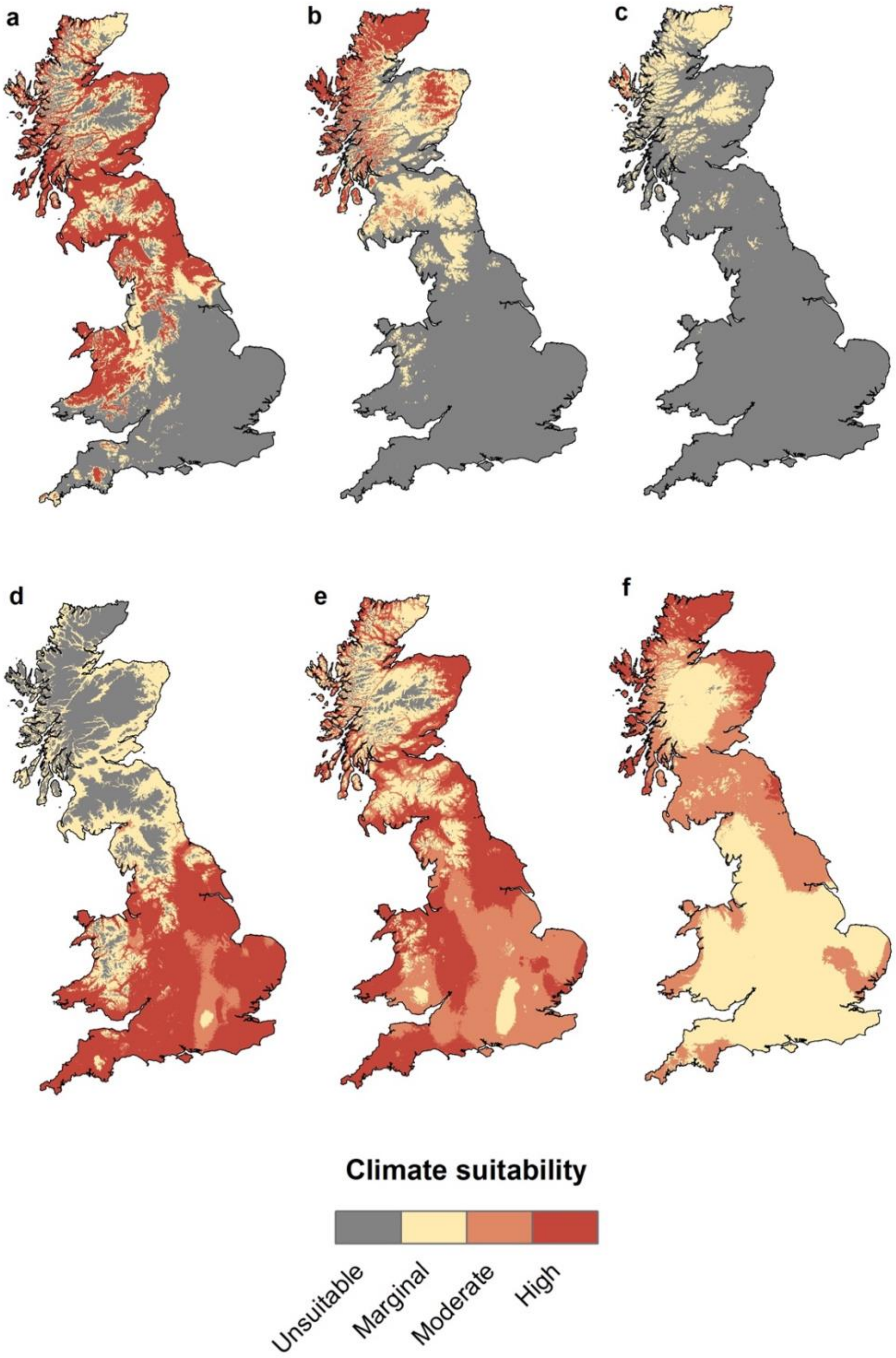

Supplementary Figure 3. Predicted climate suitability for HbS (a,b,c) and HbF (d,e,f) under current conditions (a,d) and under the optimistic (RCP 2.6; b,e) and pessimistic (RCP 8.5; c,f) future climate warming scenarios for 2070, based on climatic variables from Set 2. The future models each show consensus averaged over four GCMs. The figure was created using ArcMap (v10.8) and the Esri world countries dataset (www.esri.com).

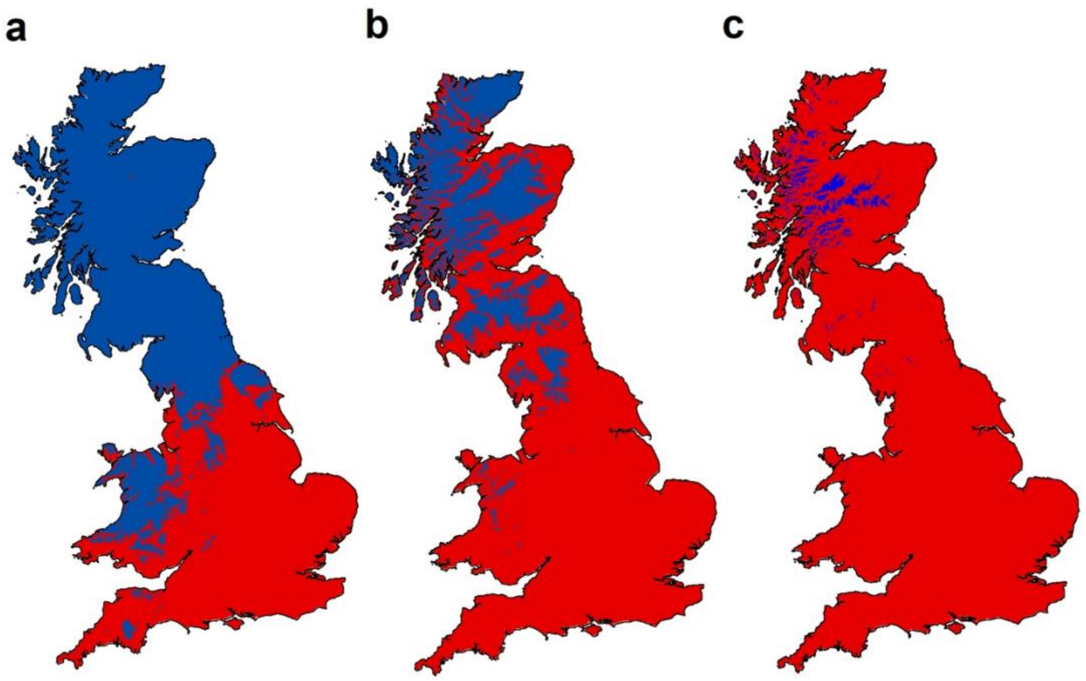

Supplementary Figure 4. The areas where the current (a) and future climate (b for RCP 2.6, c for RCP 8.5) are predicted to favour HbS over HbF (blue) and vice versa (red), based on climatic variables from Set 2. The figure was created using ArcMap (v10.8) and the Esri world countries dataset (www.esri.com).
