# Supplementary material for: Genic distribution modelling predicts adaptation of the bank vole to climate change
Source: Commun Biol. 2022 Sep 16;5:981. doi: 10.1038/s42003-022-03935-3 (PMC9481625; doi:10.1038/s42003-022-03935-3)
Supplement: Supplementary file 2 — Reporting Summary [file 42003_2022_3935_MOESM2_ESM.pdf]

nature portfolio

Corresponding author(s): Petr Kotlík
Last updated by author(s): Jul 28, 2022

# Reporting Summary

Nature Portfolio wishes to improve the reproducibility of the work that we publish. This form provides structure for consistency and transparency in reporting. For further information on Nature Portfolio policies, see our Editorial Policies and the Editorial Policy Checklist.

## Statistics

For all statistical analyses, confirm that the following items are present in the figure legend, table legend, main text, or Methods section.

| n/a | Confirmed | |
|---|---|---|
| ☐ | ☒ | The exact sample size (*n*) for each experimental group/condition, given as a discrete number and unit of measurement |
| ☐ | ☒ | A statement on whether measurements were taken from distinct samples or whether the same sample was measured repeatedly |
| ☐ | ☒ | The statistical test(s) used AND whether they are one- or two-sided<br>*Only common tests should be described solely by name; describe more complex techniques in the Methods section.* |
| ☒ | ☐ | A description of all covariates tested |
| ☒ | ☐ | A description of any assumptions or corrections, such as tests of normality and adjustment for multiple comparisons |
| ☐ | ☒ | A full description of the statistical parameters including central tendency (e.g. means) or other basic estimates (e.g. regression coefficient) AND variation (e.g. standard deviation) or associated estimates of uncertainty (e.g. confidence intervals) |
| ☐ | ☒ | For null hypothesis testing, the test statistic (e.g. *F*, *t*, *r*) with confidence intervals, effect sizes, degrees of freedom and *P* value noted<br>*Give P values as exact values whenever suitable.* |
| ☒ | ☐ | For Bayesian analysis, information on the choice of priors and Markov chain Monte Carlo settings |
| ☒ | ☐ | For hierarchical and complex designs, identification of the appropriate level for tests and full reporting of outcomes |
| ☒ | ☐ | Estimates of effect sizes (e.g. Cohen's *d*, Pearson's *r*), indicating how they were calculated |

*Our web collection on statistics for biologists contains articles on many of the points above.*

## Software and code

Policy information about availability of computer code

| | |
|---|---|
| Data collection | The present study did not use algorithms or software that are central to the research but have not yet been described in the published literature |
| Data analysis | The present study did not use algorithms or software that are central to the research but have not yet been described in the published literature |

For manuscripts utilizing custom algorithms or software that are central to the research but not yet described in published literature, software must be made available to editors and reviewers. We strongly encourage code deposition in a community repository (e.g. GitHub). See the Nature Portfolio guidelines for submitting code & software for further information.

## Data

Policy information about availability of data

All manuscripts must include a data availability statement. This statement should provide the following information, where applicable:

- Accession codes, unique identifiers, or web links for publicly available datasets
- A description of any restrictions on data availability
- For clinical datasets or third party data, please ensure that the statement adheres to our policy

The haemoglobin genotype occurrence data generated and analysed in this study are included in the supplementary information file. WorldClim climate data are publicly available at www.worldclim.org. Any additional information can be obtained directly from the authors on reasonable request.

## Human research participants

Policy information about studies involving human research participants and Sex and Gender in Research.

| | |
|---|---|
| Reporting on sex and gender | This study does not include human research participants. |
| Population characteristics | *Describe the covariate-relevant population characteristics of the human research participants (e.g. age, genotypic information, past and current diagnosis and treatment categories). If you filled out the behavioural & social sciences study design questions and have nothing to add here, write "See above."* |
| Recruitment | *Describe how participants were recruited. Outline any potential self-selection bias or other biases that may be present and how these are likely to impact results.* |
| Ethics oversight | *Identify the organization(s) that approved the study protocol.* |

Note that full information on the approval of the study protocol must also be provided in the manuscript.

# Field-specific reporting

Please select the one below that is the best fit for your research. If you are not sure, read the appropriate sections before making your selection.

☐ Life sciences ☐ Behavioural & social sciences ☒ Ecological, evolutionary & environmental sciences

For a reference copy of the document with all sections, see nature.com/documents/nr-reporting-summary-flat.pdf

# Ecological, evolutionary & environmental sciences study design

All studies must disclose on these points even when the disclosure is negative.

| | |
|---|---|
| Study description | Ecological niche models were created for two different haemoglobin types of bank voles in Britain and projected to present and future climate. A total of 50 replicates were performed for each model. |
| Research sample | The data used to train the models consisted of 40 and 57 occurrence data points for the two haemoglobin types of the bank vole in Britain. Published occurrence data (Hall 1979; Kotlík et al. 2014) were combined with new data for bank vole samples from the authors' collection. |
| Sampling strategy | Sample sizes were set according to available occurrence data and were large enough to ensure good predictive power for all models built according to previously published best practice standards (Wisz et al. 2008; Araújo et al. 2019). |
| Data collection | For haemoglobin genotyping, DNA was extracted and the beta-globin gene amplified in dedicated laboratories at the Institute of Animal Physiology and Genetics CAS and submitted to Macrogen Inc. Climate data were obtained from the WorldClim database (www.worldclim.org). |
| Timing and spatial scale | Haemoglobin data were obtained from available bank vole samples from previous studies collected largely between 1990 and 2011 in Britain. |
| Data exclusions | Filtering schemes were used for occurrence data to avoid spatial collinearity and other geographic biases; these filtering schemes were based on previously published best practice standards (Wisz et al. 2008). |
| Reproducibility | A total of 50 replicates were performed for each model to ensure reproducibility of results. Estimates were then made based on the mean of the replicates. |
| Randomization | Because we modelled climatic niches for two haemoglobin types, random assignment of samples to groups was not relevant in our study. |
| Blinding | Blinding practice was not relevant to our study because no trials were performed. |

Did the study involve field work? ☐ Yes ☒ No

# Reporting for specific materials, systems and methods

We require information from authors about some types of materials, experimental systems and methods used in many studies. Here, indicate whether each material, system or method listed is relevant to your study. If you are not sure if a list item applies to your research, read the appropriate section before selecting a response.

## Materials & experimental systems

| n/a | Involved in the study |
|---|---|
| ☒ | ☐ Antibodies |
| ☒ | ☐ Eukaryotic cell lines |
| ☒ | ☐ Palaeontology and archaeology |
| ☒ | ☐ Animals and other organisms |
| ☒ | ☐ Clinical data |
| ☒ | ☐ Dual use research of concern |

## Methods

| n/a | Involved in the study |
|---|---|
| ☒ | ☐ ChIP-seq |
| ☒ | ☐ Flow cytometry |
| ☒ | ☐ MRI-based neuroimaging |
